# Evaluation of Myeloperoxidase as Target for Host-Directed Therapy in Tuberculosis In Vivo

**DOI:** 10.3390/ijms23052554

**Published:** 2022-02-25

**Authors:** Lara C. Linnemann, Ulrich E. Schaible, Tobias K. Dallenga

**Affiliations:** 1 Division Cellular Microbiology, Research Center Borstel, 23845 Borstel, Germany; llinnemann@fz-borstel.de (L.C.L.); tdallenga@fz-borstel.de (T.K.D.); 2 German Center for Infection Research (DZIF), Site Hamburg-Lübeck-Borstel-Riems, 23845 Borstel, Germany

**Keywords:** tuberculosis, *Mycobacterium tuberculosis*, host-directed therapy, neutrophils, myeloperoxidase

## Abstract

Due to the rise of tuberculosis cases infected with multi and extensively drug-resistant *Mycobacterium tuberculosis* strains and the emergence of isolates resistant to antibiotics newly in clinical use, host-directed therapies targeting pathogenesis-associated immune pathways adjunct to antibiotics may ameliorate disease and bacterial clearance. Active tuberculosis is characterized by neutrophil-mediated lung pathology and tissue destruction. Previously, we showed that preventing *M. tuberculosis* induced necrosis in human neutrophils by inhibition of myeloperoxidase (MPO) promoted default apoptosis and subsequent control of mycobacteria by macrophages taking up the mycobacteria-infected neutrophils. To translate our findings in an in vivo model, we tested the MPO inhibitor 4-aminobenzoic acid hydrazide (ABAH) in C3HeB/FeJ mice, which are highly susceptible to *M. tuberculosis* infection manifesting in neutrophil-associated necrotic granulomas. MPO inhibition alone or as co-treatment with isoniazid, a first-line antibiotic in tuberculosis treatment, did not result in reduced bacterial burden, improved pathology, or altered infiltrating immune cell compositions. MPO inhibition failed to prevent *M. tuberculosis* induced neutrophil necrosis in C3Heb/FeJ mice in vivo as well as in murine neutrophils in vitro. In contrast to human neutrophils, murine neutrophils do not respond to *M. tuberculosis* infection in an MPO-dependent manner. Thus, the murine C3HeB/FeJ model does not fully resemble the pathomechanisms in active human tuberculosis. Consequently, murine infection models of tuberculosis are not necessarily adequate to evaluate host-directed therapies targeting neutrophils in vivo.

## 1. Introduction

With nearly half a million patients falling ill with drug-resistant tuberculosis annually and treatment success rates below 50%, multi and extensively drug-resistant strains of the *Mycobacterium tuberculosis* complex remain a threat to public health systems worldwide [1]. In certain East European and Asian countries, such as Moldova, Belarus, Russia, and Turkmenistan, among others, ≥20% of all new tuberculosis cases are drug-resistant. In those countries, ≥50% of all patients with drug-resistant tuberculosis have previously been treated for tuberculosis, suggesting preceding insufficient treatment or failure. Multi and extensively drug-resistant tuberculosis needs to be treated with combinations of second- and third-line drugs, such as Moxifloxacin and ethionamide, often accompanied by severe side effects, including irreversible peripheral neuropathy and hearing loss, renal and liver toxicity, epileptic seizures, and psychosis [2,3]. In the past, *M. tuberculosis* has been shown to quickly acquire resistances even to newly developed and last-resort antibiotics within a few years after their introduction into the clinics as well as the occurrence of so-called totally drug-resistant strains [4,5]. These observations together with the host response-driven pathogenesis of active tuberculosis emphasize the need to explore alternative treatment strategies adjunct to antibiotics.

Host-directed therapies for tuberculosis target immune and inflammatory mechanisms involved in pathogenesis and hampering protective immunity. Massive influx of neutrophils to the pulmonary sites of *M. tuberculosis* infection, their activation, degranulation, oxidative burst, and subsequent necrotic cell death have been associated with dysregulated, over-reacting host responses characterized by exacerbated inflammation, cytokine storms, tissue damage leading to long-term pathological sequelae, and contagious coughing [6,7,8,9,10]. We previously described that human neutrophils quickly succumb to necrotic cell death upon *M. tuberculosis* infection in vitro. Subsequent uptake of infected necrotic neutrophil remnants by macrophages—a process termed necrophorocytosis—promotes mycobacterial replication in these more long-lived host cells [11,12]. As a result, however, these macrophages become infected and subsequently undergo necrotic cell death after a few days, thereby releasing a multiplicity of mycobacteria that are ready to infect other host cells. Thus, a vicious circle in host cell necrosis is established that likely takes place in the lungs of active tuberculosis patients with exacerbated inflammation [9]. Mycobacteria-driven neutrophil necrosis depends on reactive oxygen species and can be prevented by pharmacological inhibition of MPO resetting the neutrophil cell death pathway to default apoptosis. When macrophages take up infected apoptotic neutrophils, they are able to control the growth of *M. tuberculosis*, which is associated with anti-mycobacterial apoptotic cell death. Thereby, the vicious circle is interrupted. In mouse models of tuberculosis, increased neutrophil infiltration was accompanied by exacerbated pathogenesis [13,14]. Studies targeting neutrophils have shown that blocking recruitment of or depleting neutrophils ameliorate experimental tuberculosis [15,16]. Therefore, we explored the sensitivity of neutrophil MPO to ameliorate experimental tuberculosis in the susceptible C3HeB/FeJ mouse model in vivo. These mice develop pathologies similar to those in human active tuberculosis patients [17]. Here, we used the MPO inhibitor 4-Aminobenzoic acid hydrazide (ABAH) that has been previously shown in other studies to be beneficial against different disease models in vivo, such as experimental autoimmune encephalomyelitis and ischemic stroke [18,19,20].

Herein we report that ABAH treatment, alone or in combination with isoniazid, of C3HeB/FeJ mice experimentally infected with *M. tuberculosis* did not alter bacterial burden, compositions of immune cell infiltrates, cytokine responses, or pathological sequelae, nor did inhibition of MPO using ABAH prevent neutrophil necrosis in vitro. The relevance of MPO-mediated necrotic cell death for tuberculosis pathogenesis may differ between humans and mice and requires future studies and distinct tuberculosis models to reveal whether MPO remains a valid target for host-directed therapy in humans.

## 2. Results

### 2.1. Mycobacterial Burden and Disease Manifestations in M. tuberculosis Infected C3Heb/FeJ Mice Remain Unaffected by ABAH Treatment

**Figure 1:**
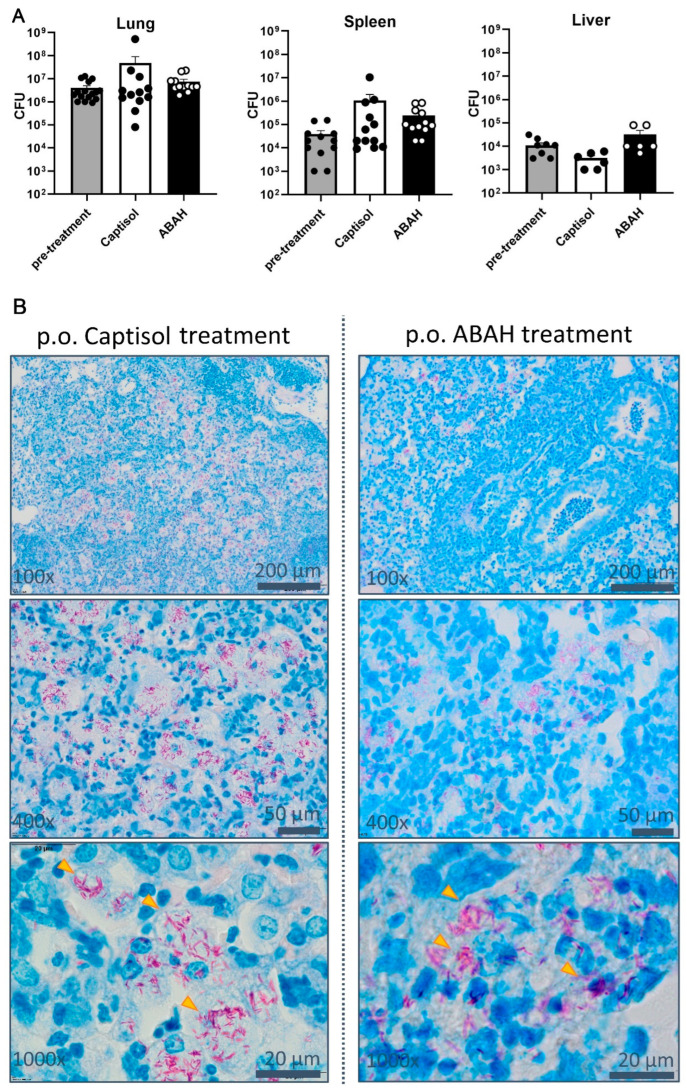
MPO inhibition did not reduce mycobacterial numbers or inflammatory infiltrates in infected mice. C3HeB/FeJ mice were aerosol-infected with 100–150 CFUs H37Rv, and bacterial burdens were calculated by CFU analysis at 25 dpi (pre-treatment) or 35 dpi (Captisol^®^ (San Diego, CA, USA), ABAH) for lungs, spleens, and livers (**A**). Depicted are data from two independent experiments pooled in one graph. Error bars indicate SEM, ordinary one-way ANOVA with Tukey’s multiple comparisons test. Ziehl–Neelsen stainings counterstained with Hemalaun showed no differences in inflammatory infiltrates and mycobacterial distribution (arrowheads) within lungs of *M. tuberculosis* infected mock- vs. ABAH-treated C3HeB/FeJ mice (**B**). Lungs were removed at day 35, embedded in paraffin, and stained. Depicted are representative pictures.

Based on our previous in vitro findings in human neutrophils that inhibition of MPO limits *M. tuberculosis* induced and MPO-mediated necrotic cell death in vitro and promotes control of mycobacterial growth, we hypothesized that ABAH treatment can also be beneficial in vivo against experimental tuberculosis in susceptible C3HeB/FeJ mice where neutrophils are involved in disease exacerbation. We confirmed our previous findings that human neutrophils undergo a necrotic-like cell death upon *M. tuberculosis* H37Rv infection which is preventable by MPO inhibition (Supplementary Figure S1A). The presence of MPO in infected lungs from C3HeB/FeJ mice was revealed by immunohistology showing MPO-positive cells within inflammatory infiltrates (Supplementary Figure S1B, arrows). Notably, MPO staining was not restricted to neutrophils, distinguishable by their polymorphonuclear morphology, but was also seen in other cells resembling the morphologies of macrophages due to their relatively large cytoplasm and large and decondensed nuclei. Thus, target cells for MPO inhibition by ABAH were present at sites of infection. C3HeB/FeJ mice were infected with *M. tuberculosis* H37Rv and treated with Captisol^®^ vehicle or ABAH twice a day from day 25 post infection on until day 35 (Supplementary Figure S2). Highest mycobacterial burden was found in the lungs of Captisol^®^ vehicle treated mice at day 35 post infection (Figure 1A). Although MPO inhibitor treatment slightly reduced the bacterial burden, this difference was not statistically significant. Similar results were found for mycobacterial burdens of spleens and livers (Figure 1A). Monitoring weight loss and scoring the health status of infected mice did not reveal significant differences between vehicle- and ABAH-treated groups (Supplementary Figure S3). These observations were further confirmed by histopathological analysis of the lungs. Combined Ziehl–Neelsen and Hemalaun stainings of lung tissue sections revealed similar numbers, areas, and appearances of inflammatory lesions (Supplementary Figure S4A). MPO-positive polymorphonuclear cells were found within those infiltrates, but there were no gross differences neither in distribution nor numbers of MPO-positive cells between vehicle- and ABAH-treated groups (Supplementary Figure S4B, arrowheads). Higher magnifications of Ziehl–Neelsen stained sections showed similarly structured lesions containing *M. tuberculosis* clusters (Figure 1B, arrowheads). *M. tuberculosis* appeared to be intracellular in intact cells as well as extracellularly embedded in material resembling cellular necrotic debris.

Taken together, although MPO-positive neutrophils and *M. tuberculosis* clusters were present in lungs of infected C3HeB/FeJ mice, we did not observe any changes regarding bacterial burden, clinical parameters (weight, health score), and lung pathology upon MPO inhibitor treatment twice per day between days 25 and 35 post infection.

### 2.2. Quantitative Characterization of Cellular Compositions and Effector Functions in Lungs of M. tuberculosis Infected Mice with or without ABAH Treatment

**Figure 2:**
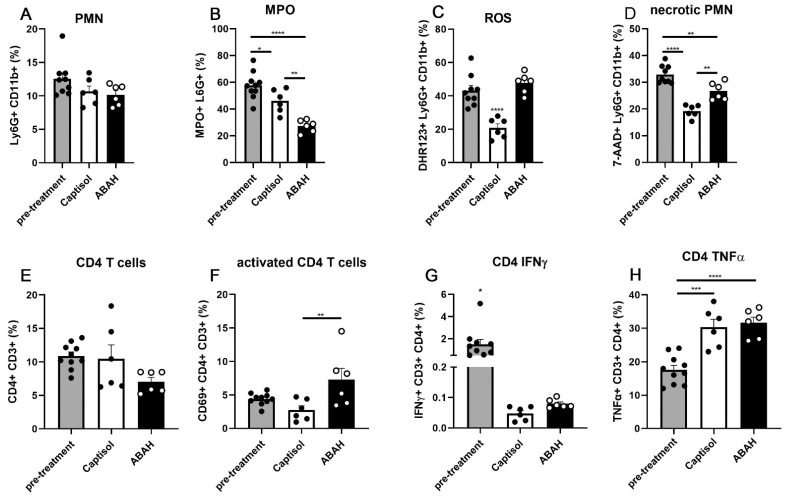
Characterization of neutrophils and T cells in lungs of *M. tuberculosis* infected mice. C3HeB/FeJ mice were aerosol-infected with 100–150 CFUs H37Rv, and single-cell suspensions of the lungs were prepared at 25 dpi (pre-treatment) or 35 dpi (Captisol, ABAH), labeled with indicated markers, and analyzed by flow cytometry. Neutrophil numbers did not change between the groups (**A**). MPO expression of neutrophils was reduced upon ABAH treatment (**B**). Rates of ROS production by and necrosis of neutrophils were reduced upon mock treatment (**C**,**D**). Frequencies of CD4 T cells were similar in all groups (**E**) while activated ones were more numerous in the ABAH-treated group (**F**). For intracellular cytokine staining, cells were re-stimulated with CD3/CD28 (**G**,**H**). Before treatment start at 25 dpi, frequencies of IFN-g-producing CD4 T cells were higher and those of TNF-a-producing CD4 T cells were lower when compared to the mock- and ABAH-treated groups at 35 dpi. Gating strategy for flow cytometry in Supplementary Figure S5. Depicted is one experiment with 6–10 mice per group. Error bars indicate SEM, ordinary one-way ANOVA with Tukey’s multiple comparisons test. *: *p* ≤ 0.05, **: *p* ≤ 0.01, ***: *p* ≤ 0.005, ****: *p* ≤ 0.001.

Next, we quantified the cellular composition of lung infiltrating cells, their effector functions, and necrosis rates with a focus on pulmonary neutrophils from vehicle- vs. ABAH-treated infected C3HeB/FeJ mice. Upon pulmonary *M. tuberculosis* infection, these mice have been described to develop a PMN-driven, proinflammatory, necrotic granulomatous phenotype [17,21]. Thus, the presence of MPO-expressing PMN, ROS production, and necrosis indicate the pathological state of necrotizing lung lesions, which was further assessed by quantitative flow cytometry of whole lung cells. After debris and doublet cell exclusion (for gating strategy, see Supplementary Figure S5), neutrophils were identified as CD3^−^ Ly6G^+^ CD11b^+^ cells. No significant differences in neutrophil frequencies were found within lungs 25 d post infection before treatment start or upon vehicle-only or ABAH treatment 35 d post infection (Figure 2A). Population frequencies are shown as percentage of all CD45+ cells. Of note, using total cell counts did not show any differences when compared to frequencies (percentage of CD45+) in any analyzed populations (data not shown). Interestingly, frequencies of MPO^+^ neutrophils significantly decreased between day 25 and day 35 post infection but further decreased upon ABAH treatment when compared to the vehicle-treated group (Figure 2B). With disease progression between day 25 (pre-treatment) and 35 post infection (vehicle treatment only), neutrophils showed reduced ROS production over time (Figure 2C) as assessed by membrane-permeable Dihydrorhodamine 123 (DHR123) staining that becomes fluorescent upon oxidation. Surprisingly, ABAH treatment did not lead to a reduction in ROS^+^ neutrophil numbers when compared to the vehicle-treated group but significantly increased frequencies of these cells to a similar level as in the pre-treatment group. Frequencies of necrotic PMN were also significantly enhanced in lungs of ABAH-treated mice when compared to vehicle-treated ones (Figure 2D). Of note, frequencies of MPO^+^ ROS^+^ necrotic neutrophils were always higher in animals at day 25 post infection before treatment started.

Since IFN-g-producing CD4^+^ cells are considered pivotal in protection against *M. tuberculosis* infection, we checked their frequencies and phenotypes. Frequencies of CD4 T cells within the lungs of all three groups were similar (Figure 2E). Frequencies of activated CD4 T cells, as determined by CD69 expression [22], were significantly increased in lungs of ABAH-treated mice (Figure 2F). Presence of IFN-g^+^ CD4 T cells decreased over time between day 25 and 35 post infection, while TNF-a^+^ ones increased, but no differences between vehicle- and ABAH-treated mice were observed (Figure 2G,H). Similarly, frequencies of IFN-g^+^ CD8 T cells also decreased with disease progression but were similar in both treatment groups at day 35 post infection (Supplementary Figure S6A). Frequencies of TNF-a^+^ CD8 T cells and IFN-g^+^ NK T cells, as characterized by CD49b^+^ CD3^+^ staining, were similar in all groups (Supplementary Figure S6B,C). Frequencies of TNF-a^+^ NK T cells increased from day 25 post infection to day 35 post infection while no differences were found between the vehicle- and the ABAH-treated groups (Supplementary Figure S6D).

Frequencies of CD3^−^ CD49b^+^ NK cells decreased between day 25 and day 35 post infection, but no difference between vehicle- and ABAH-treated groups was observed (Supplementary Figure S6E). We differentiated between dendritic cells and interstitial and alveolar macrophages as described before [23]. Frequencies of CD11b^+^/CD11c^+^/MHCII^+^ dendritic cells increased over time independent of the treatment (Supplementary Figure S6F). Frequencies of CD11b^+^ CD11c^−^ MHCII^int^ cells were significantly lower in lungs of vehicle-treated mice at day 35 post infection, while those of ABAH-treated mice were similar to the pre-treatment group (day 25 post infection) (Supplementary Figure S6G). This population includes interstitial macrophages and eosinophils. Frequencies of CD11b^−^ CD11c^−^ MHCII^int^ alveolar macrophages were highest at day 25 post infection before treatment start (Supplementary Figure S6H) but did not differ between the treatment groups at day 35 post infection.

Taken together, although ABAH treatment of infected mice resulted in decreased frequencies of MPO^+^ neutrophils within the lungs, it neither led to reduced neutrophil-associated ROS production nor necrosis rates. Overall frequencies of neutrophils, T cells, DCs, and alveolar macrophages were not altered by ABAH treatment. Notably, frequencies of activated CD4^+^ T cells were significantly increased in lungs of ABAH-treated animals.

### 2.3. Necrosis and MPO within M. tuberculosis Lungs Were Not Affected by ABAH Treatment

To quantify overall tissue necrosis in *M. tuberculosis* infected lungs, lactate dehydrogenase (LDH) activities within the extracellular protein fractions were measured. LDH is a strictly intracellular protein, and its extracellular presence can be used as a marker for necrotic cell death events associated with plasma membrane rupture. Extracellular protein fractions were isolated from pulmonary single-cell suspensions by incubation with protein extraction buffer and acetone precipitation. The highest concentration of LDH was found before treatment start at day 25 post infection, while activities were significantly lower at day 35 (Supplementary Figure S7). However, no differences were detected between the vehicle- and the ABAH-treated groups.

**Figure 3:**
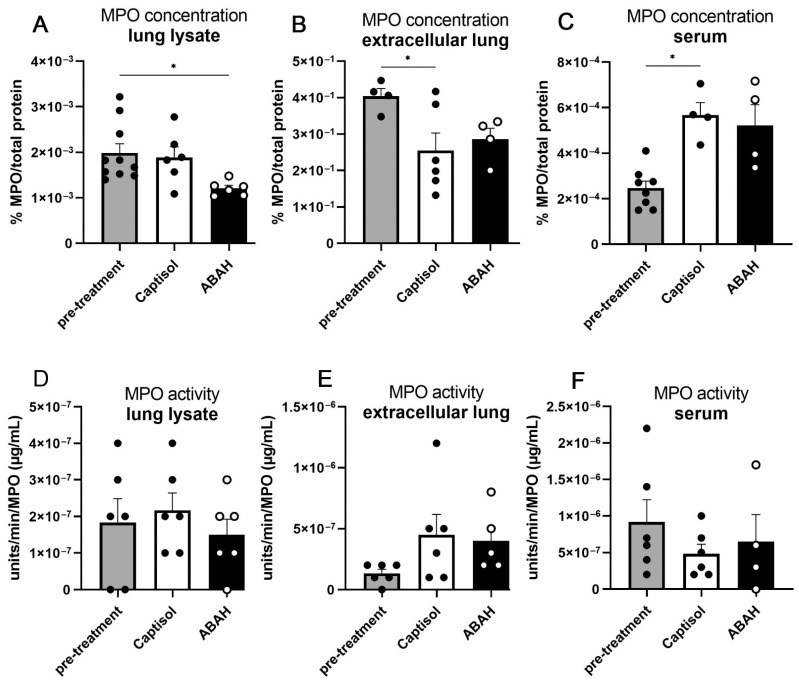
No significant changes in MPO concentration and activity upon ABAH treatment (**A**–**F**). C3HeB/FeJ mice were aerosol-infected with 100–150 CFUs H37Rv, and MPO concentrations and activities of indicated samples were assessed using ELISA or colorimetric analysis, respectively, at 25 dpi (pre-treatment) or 35 dpi (Captisol, ABAH). Depicted is one experiment with 6–10 mice per group. Error bars indicate SEM, ordinary one-way ANOVA with Tukey’s multiple comparisons test, * *p* ≤ 0.05.

Next, we determined MPO protein concentration and enzymatic activity in extracellular protein fractions versus total lung lysates and serum. MPO concentrations in whole lung lysates did not change between day 25 and day 35 post infection in the solvent-treated group and were slightly reduced in the ABAH-treated group (Figure 3A). At 35 days post infection, less MPO was measured in extracellular lung fractions, but more MPO was found in serum compared to 25 days post infection (Figure 3B,C). However, no differences were observed between both treatment groups. Interestingly, average MPO protein concentrations normalized to total protein content were lowest in serum samples, intermediate in total lung lysates, and highest in extracellular protein fractions.

Taken together, MPO inhibition did not affect MPO activity in whole lung lysates or extracellular protein fractions or serum samples (Figure 3D–F). We observed similar levels of lung tissue necrosis and MPO concentrations and activities in vehicle- vs. ABAH-treated mice.

### 2.4. Slight Reductions in Proinflammatory Cytokine Responses in ABAH-Treated Lungs

**Figure 4:**
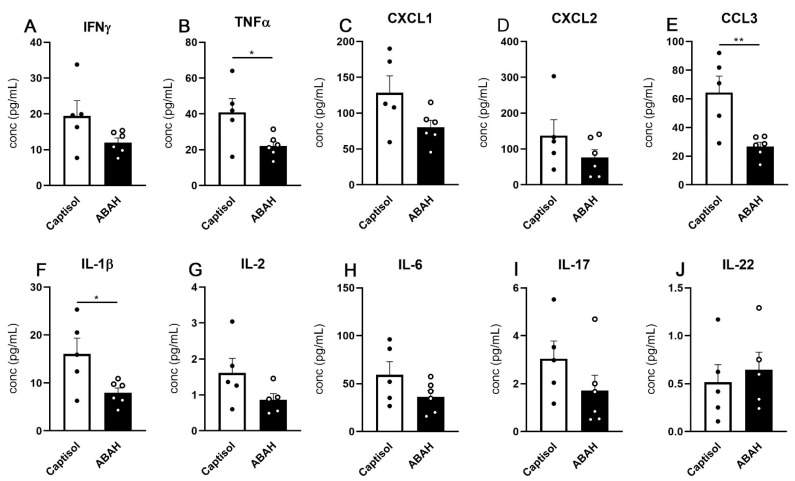
Cytokine responses in ABAH-treated mice. C3HeB/FeJ mice were aerosol-infected with 100–150 CFUs H37Rv, and cytokines were measured using a multiplex immunoassay in lung lysates at 35 dpi. Concentrations of all analyzed cytokines tended to be lower in ABAH-treated mice (**A**–**I**), except for IL-22 (**J**), but only TNF-a, CCL3, and IL-1β showed significant reductions. Depicted is one experiment with 5–6 mice per group. Error bars indicate SEM, unpaired t test. *: *p* ≤ 0,05, **: *p* ≤ 0,01.

Next, we evaluated the cyto- and chemokine responses in whole lung lysates of vehicle- vs. ABAH-treated mice at day 35 post infection. Upon ABAH treatment, reduced concentrations of the proinflammatory cytokines IFN-g, TNF-a, IL-1b, IL-17, and IL-2, the immunoregulatory IL-6, and the neutrophil attractors CXCL1, CXCL2, and CCL3, but not of the inflammation-resolving IL-22, were observed; however, only TNF-a, CCL3, and IL-1β concentrations were reduced in ABAH-treated mice in a statistically significant manner (Figure 4A–J). Slightly reduced concentrations of the proinflammatory leukocyte attractors CCL2, CCL4, CCL5, CCL20, CCL22, CXCL9, and CXCL10, the neutrophil attractor and activators CCL3 and CXCL5, eosinophil attractor CCL11, T-cell attractor CCL17, and B-cell attractor CXCL13 were also observed in lungs of ABAH-treated animals but not in a statistically significant manner (Supplementary Figure S8). Taken together, ABAH treatment of *M. tuberculosis* infected C3HeB/FeJ mice led to slightly reduced inflammatory cyto- and chemokine concentrations in the lungs.

### 2.5. Co-Treatment with ABAH and Isoniazid

**Figure 5:**
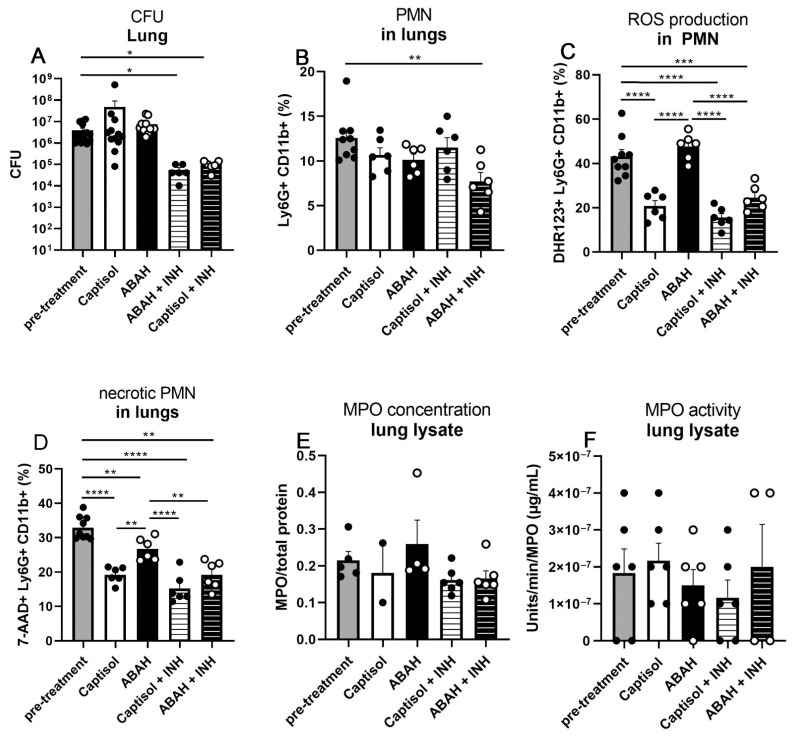
Isoniazid and ABAH co-treatment in mice. C3HeB/FeJ mice were aerosol-infected with 100–150 CFUs H37Rv and analyzed at 25 dpi (pre-treatment) or 35 dpi (all other groups). Co-treatment with a suboptimal concentration of isoniazid and ABAH did not lead to synergistic effects regarding the reduction of bacterial burden assessed by CFU analysis in lung lysates (**A**), MPO concentration and activity in lung lysates measured by ELISA (**B**), and colorimetric assays (**C**), respectively, and number of ROS+, necrotic, and overall neutrophils within lungs quantified by flow cytometry after indicated labeling of cells (**D**–**F**) when compared to the mock- and isoniazid-treated control group (Captisol + INH). Bars for the groups pre-treatment, Captisol, and ABAH represent the same data sets used in Figure 1, Figure 3 and Figure 4. Gating strategy for flow cytometry in Supplementary Figure S5. Depicted is one experiment with 6–10 mice per group. Error bars indicate SEM, ordinary one-way ANOVA with Tukey’s multiple comparisons test. *: *p* ≤ 0.05, **: *p* ≤ 0.01, ***: *p* ≤ 0.005, ****: *p* ≤ 0.001.

To study combined effects of ABAH co-treated with low doses of the first-line antibiotic isoniazid (INH), animal experiments and key read-out assays were performed. No differences in weight loss and health scores were observed between the vehicle- and the ABAH-INH co-treated groups (Supplementary Figure S9). Low-dose treatment with INH for 10 days between day 25 (pre-treatment group) and 35 post infection already reduced pulmonary bacterial burdens (Figure 5A). However, co-administration of ABAH with INH neither further reduced the bacterial loads nor altered MPO concentrations and activities, ROS generation, and neutrophil necrosis induction (Figure 5A–E). However, ABAH co-treatment with INH further reduced frequencies of neutrophils in lungs when compared to those treated with INH alone (Figure 5F). Despite high frequencies of neutrophils present in lungs of INH-only treated animals (Figure 5F), ROS production and neutrophil necrosis were also significantly reduced in ABAH co-treated mice (Figure 5D). Taken together, co-treatment with ABAH + INH did not result in improved outcomes compared to INH alone.

### 2.6. MPO Inhibition Did Not Reduce ROS Production and Necrosis in Infected Murine Neutrophils In Vitro

To further assess why MPO inhibition by ABAH did not reduce bacterial burden, tissue pathology, or cellular necrotic rates, we studied isolated neutrophils from C3HeB/FeJ mice. Neutrophils were isolated from bone marrow, matured in vitro by incubation with G-CSF for 48 h [24,25], and infected with *M. tuberculosis* before MPO concentrations, MPO activities, ROS production, and necrosis rates were analyzed.

**Figure 6:**
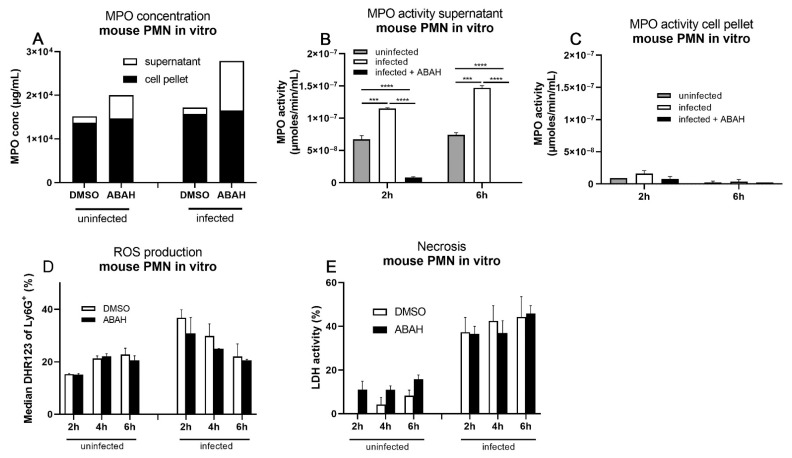
Failure to reduce MPO concentration, ROS production, and necrosis upon MPO inhibition and *M. tuberculosis* infection in bone-marrow-derived mouse neutrophils in vitro. C3HeB/FeJ bone marrow cells were cultivated for 2 d at the presence of rG-CSF and infected with Mtb at MOI 3. (**A**) MPO concentrations were quantified by ELISA, (**B**,**C**) MPO activity was colorimetrically measured using an artificial substrate (TMB), (**D**) intracellular ROS production was determined by DHR123 fluorescence using flow cytometry, and (**E**) necrosis rates were assessed by quantification of extracellular LDH activity. Gating strategy for flow cytometry in Supplementary Figure S5. Depicted is one experiment (**A**) or two independent experiments pooled in one graph (**B**–**E**). Error bars indicate SEM, two-way ANOVA with Sidak’s multiple comparisons test. ***: *p* ≤ 0.005, ****: *p* ≤ 0.001.

MPO concentrations in supernatants were higher in neutrophil cultures treated with ABAH regardless of whether they were infected or left uninfected (Figure 6A). No differences in cellular MPO concentrations were observed. MPO enzyme activities in neutrophil supernatants significantly increased upon infection but were abolished by ABAH treatment (Figure 6B), indicating that *M. tuberculosis* infection induced release of active MPO, which is inhibited by ABAH treatment. Intracellular MPO activities were generally very low in all groups tested and were not affected by ABAH treatment (Figure 6C). *M. tuberculosis* infection of neutrophils led to increased intracellular ROS concentrations when compared to uninfected cells (Figure 6D) as assessed by intracellular DHR123 staining and flow cytometry. However, ABAH treatment reduced intracellular ROS levels only slightly but not significantly upon infection. This is in line with the observation that mainly extracellular MPO activity is affected by infection and subsequent ABAH treatment. Importantly, *M. tuberculosis* infection quickly induced necrosis in murine neutrophils in vitro (Figure 6E) in a similar manner to human ones [11]. However, in contrast to human neutrophils, ABAH treatment failed to reduce necrotic cell death rates in mouse neutrophils in vitro (Figure 6E). Differential effects of ABAH treatment between human and mouse neutrophils may explain why ABAH did not improve the outcome of infection in C3HeB/FeJ mice in vivo alone or together with INH.

Taken together, treatment of susceptible C3HeB/FeJ mice infected with *M. tuberculosis* H37Rv with the MPO inhibitor ABAH alone or with INH did not improve bacterial burden, pathology, body weight, health score, tissue necrosis, neutrophil infiltration, neutrophil necrosis, or ROS production. The failure of MPO inhibition to alter *M. tuberculosis* outcome in vivo is corroborated by our finding that *M. tuberculosis* infection induced necrotic cell death of C3HeB/FeJ bone-marrow-derived neutrophils cannot be prevented in vitro by ABAH treatment. This is in stark contrast to our previous results from human blood-derived neutrophils [11,12] indicating that necrosis induction by mycobacteria in murine neutrophils is independent of MPO.

## 3. Discussion

As previously reported, *M. tuberculosis* induced necrotic cell death of human neutrophils can be prevented by inhibition of MPO using ABAH [11,12], which promotes downstream control of intracellular mycobacterial growth by human macrophages upon uptake of infected neutrophils. Here, we aimed to re-translate our in vitro findings in human cells into the in vivo mouse model of experimental tuberculosis. In contrast to the commonly used tuberculosis models in C57BL/6 (BL/6) and BALB/c mice, *M. tuberculosis* infection in C3Heb/FeJ mice reflects, to a certain degree, the pathology of human tuberculosis, including development of central necrotic granulomas and neutrophil influx. Extracellularly generated hypochlorous acid, the main ROS produced by MPO, has been shown to cause severe tissue, cellular, and DNA damage to bystander cells, which propagate disease in models of subacute and ischemic stroke, multiple sclerosis, and inflammatory bowel disease [18,19,20,26,27,28,29,30]. Indeed, ABAH treatment resulted in improved clinical parameters in murine models of stroke and experimental autoimmune encephalomyelitis [19,31,32]. Thus, we employed the murine C3Heb/FeJ model of tuberculosis to study whether MPO inhibition by ABAH can ameliorate tuberculosis pathogenesis in vivo.

Similar to human neutrophils, bone-marrow-derived Ce3HeB/FeJ neutrophils quickly succumbed to a necrotic cell death in vitro upon *M. tuberculosis* infection. However, necrotic cell death could not be prevented by MPO inhibition even though extracellular MPO activity was virtually abolished. This fundamental difference between murine and human neutrophils in their in vitro response to MPO inhibition may represent the reason why ABAH alone or in combination with INH did not improve disease outcome in *M. tuberculosis* infected C3HeB/FeJ mice.

In mouse strains differentially susceptible or resistant to *M. tuberculosis* infection, enhanced susceptibility correlates with increased neutrophil influx into lungs [13,14,16]. In human pulmonary samples, neutrophils represent the predominant cell population carrying the main mycobacterial load [10]. Disease progression has been associated with a neutrophil-dominated blood transcriptomic signature, and exacerbated pathology is thought to be neutrophil-driven [8,33,34,35]. Compared to human tuberculosis, we observed low frequencies of neutrophils in infected lungs of C3HeB/FeJ mice (~10–13%, Figure 2A). Murine cells have been reported to have a strikingly lower MPO activity of 20 compared to 109 Units/5 × 10^6^ cells in human ones [36]. Notably, this observation was made in BL/6 but not in C3HeB/FeJ mice. The five-time reduction in MPO activity in murine cells can explain why MPO inhibition by ABAH did not lead to an overall reduction in ROS production since the contribution of MPO-generated to total ROS may be small. While MPO plays a major role in *M. tuberculosis* infected human neutrophils, its contribution to the oxidative burst in murine neutrophils upon *M. tuberculosis* infection may be lower. Other ROS, such as NADPH-oxidase-generated super oxide, may be more relevant for necrotic cell death of murine cells. More importantly, in murine tuberculosis models, reactive nitrogen species (RNS) have been assigned to a more important role in anti-mycobacterial responses than ROS. Genetic disruption of the essential catalytic gp91^phox^ or NOX2 subunit of NADPH oxidase in mice had only minor effects on mycobacterial loads and disease outcome early in infection. In contrast, mice deficient in inducible NO synthase (NOS2) displayed exacerbated mycobacterial growth and reduced survival [37,38]. In vitro stimulation of gp91^phox^-deficient macrophages with IFN-g restored their ability to kill *M. tuberculosis* but in an RNS-dependent manner [37]. NOS2-deficient macrophages were unable to contain the infection despite IFN-g stimulation. It should, however, be noted that the gene knockout mice employed in these studies had the genetic C57BL/6 background associated with resistance to *M. tuberculosis* infection. The contribution of RNS vs. ROS in C3HeB/FeJ to either protective host responses or disease progression has not yet been studied. More importantly, the questions of whether murine neutrophils contribute either to protective RNS or disease-promoting ROS production and whether neutrophils from resistant C57BL/6 differ from those of susceptible C3HeB/FeJ mice need to be a focus of future studies. It should be noted that IFN-g-induced RNS production promotes apoptotic cell death of infected macrophages contributing to elimination of mycobacteria (Herbst et al., 2011).

On a global level, less neutrophils in inflammatory infiltrates of the lungs, less MPO activity in these neutrophils, and less ROS-mediated resistance toward *M. tuberculosis* in mice as compared to humans may limit the effects of MPO inhibition in experimental murine tuberculosis and the usability of these mice to evaluate MPO-targeting therapies.

It has been recently shown that infiltrating neutrophils in C3Heb/FeJ mice upon infection with the hypervirulent clinical isolate HN878 induce NETosis and the formation of NETs [39]. HN878 belongs to lineage 2 (Beijing) and is considered hypervirulent regarding its growth in vivo and animal survival rates upon infection. The lab reference strain H37Rv used in this study belongs to lineage 4 (Euro-American) and does not display hypervirulent features. In this study, we did not specifically look for NETosis but rather for general necrotic-like cell death as defined by plasma membrane leakage and spill of intracellular molecules into the extracellular space, which includes NETosis. MPO activity can be essential for NETosis induction depending on the stimulus [40]. As shown in here, MPO inhibition by ABAH did not reduce overall necrotic cell death rates upon infection with *M. tuberculosis* in vitro as determined by extracellular lactate dehydrogenase activity (Figure 6E). Thus, we can exclude effects of MPO inhibition on NETosis rates. As another source of ROS and released DNA, eosinophils have recently been reported to be part of the innate immune response in murine experimental tuberculosis [41]. Eosinophils carry the eosinophil peroxidase, a homolog to the neutrophil MPO, that catalyzes the reaction of H_2_O_2_ and halide ions, e.g., bromide, to produce hypohalous acid [42]. Similarly to neutrophils, eosinophils are also capable of producing extracellular traps from released DNA [43]. Thus, it cannot be excluded that inhibition of neutrophil MPO by ABAH had effects on eosinophilic numbers, eosinophil peroxidase, or the formation of eosinophilic extracellular traps. However, overall necrotic-like events as detected by extracellular lactate dehydrogenase activity within lungs did not show any differences between the treated and the control group (Supplementary Figure S7), which suggests that eosinophil trap formation is also similar in both experimental groups.

In this study, we used DHR123 to detect ROS production by flow cytometry. DHR123 diffuses into the cell by crossing the plasma membrane and translocates into mitochondria where it becomes fluorescent upon oxidation. Thus, we were only able to measure intracellular while missing extracellular ROS production. However, extracellular MPO may be more relevant for neutrophil necrotic cell death induction since only the extracellular enzyme concentration and activity were enhanced upon *M. tuberculosis* infection, while ABAH only diminished extracellular MPO activity (Figure 6). With respect to the prominent role of RNS in mycobacterial killing in mice, it should be mentioned that DHR123 also becomes fluorescent upon oxidation by peroxynitrite, the reaction product of NO with super oxide in vivo [44,45]. Thus, DHR123 can be seen as a general indicator of reactive nitrogen oxide species [46,47]. However, DHR123 would not signal upon nitrosylation by RNS alone since upstream ROS are still required to generate peroxynitrite. Though blood neutrophils are not readily equipped with NOS2, it has been shown that they are able to produce NO via NOS2 under different pathological conditions, including bacterial infections [48,49,50]. Thus, RNI may have overshadowed the effects of ROS, especially of those minor MPO-derived ones.

C3HeB/FeJ bone-marrow-derived neutrophils exhibited increased MPO activity and ROS production upon *M. tuberculosis* infection in vitro and eventually succumbed to necrotic cell death, which reflected the situation of human neutrophils [11,12]. Although MPO activity could be abolished when applying ABAH, overall ROS production was not significantly decreased (Figure 6B,D). This observation suggests that in contrast to human neutrophils, murine C3HeB/FeJ neutrophils can produce ROS from other sources than MPO upon *M. tuberculosis* infection. In turn, MPO inhibition cannot prevent neutrophil necrosis. Both failure of reducing ROS and subsequently preventing necrosis may provide an explanation for the failure of MPO inhibition to ameliorate the outcome of experimental tuberculosis in vivo in C3Heb/FeJ mice. It should be noted that in the relatively little-studied C3HeB/FeJ mouse model, other potential differences regarding function and distribution of macrophages and neutrophils, NADPH oxidase, NOS2 and RNS, and MPO were not investigated in this study but may differently control pathogenesis in humans vs. C3HeB/FeJ mice.

Taken together, translation into a murine in vivo model of our previous observation that *M. tuberculosis* induced neutrophil necrosis can be prevented by MPO inhibition revealed substantial differences between mouse and human neutrophils. In the murine system, MPO inhibition had no effect on *M. tuberculosis* induced neutrophil necrotic cell death, granuloma necrosis, health score, body weight, and, ultimately, bacterial burden in vivo. Co-treatment of ABAH together with isoniazid neither supported the anti-bacterial effect of the antibiotic nor improved lung pathology when compared to isoniazid treatment alone.

Based on the differences observed between human and murine neutrophils, we cannot exclude the fact that MPO inhibition may have a beneficial host-directed therapeutic effect in human tuberculosis. This raises the question of to what extent the pathogenesis of experimental tuberculosis in C3HeB/FeJ mice is comparable to the situation in humans with active disease. Ultimately, *M. tuberculosis* induced necrotic cell death of murine neutrophils was neither MPO- nor hypochlorous-acid-dependent. This fundamental difference should be considered when using the C3HeB/FeJ mouse as a model for tuberculosis to design neutrophil-targeting host-directed therapies. Other animal models that have been reported to reflect human tuberculosis pathology, such as guinea pigs, rabbits, and non-human primates, may be more appropriate to evaluate pharmacological interventions of neutrophil-associated pathomechanisms in experimental tuberculosis.

## 4. Materials and Methods

### 4.1. Ethical Statements

Experimental work with human peripheral blood cells of healthy volunteers was approved by the ethical committee of the University of Lübeck, Germany (No. 22-202A), and written consents were obtained. Animal experiments were approved by the ethics committee of the competent authority of Germany (No. V 244-16731/2020(58-5/17)).

### 4.2. Isolation of Human Neutrophils

Human peripheral blood neutrophils were isolated as described before [51]. In brief, 20 mL of pre-warmed (37 °C) Histopaque (Sigma-Aldrich, St. Louis, MO, USA) was overlayered with 30 mL blood. After density centrifugation at 800× *g* at RT for 20 min, cells were layered onto a discontinuous Percoll (Sigma-Aldrich) density gradient (85%, 80%, 75%, 70%, 65%) and centrifuged at 800× *g* at RT. Neutrophils were incubated at 37 °C with 5% CO_2_. Purities of neutrophil cultures were >98% as assessed by Haematoxylin & Eosin and Giemsa staining.

### 4.3. M. tuberculosis Culture

Frozen aliquots of the mycobacterial strain H37Rv were thawed and cultured at 37 °C in Middlebrook 7H9 broth (BD Biosciences, Heidelberg, Germany) supplemented with 0.05% Tween 80 (Sigma-Aldrich, St. Louis, MO, USA) and 10% OADC (Oleic acid, Albumin, Dextrose, Catalase) (BD Biosciences, Heidelberg, Germany). At mid-log phase of growth, a part of the culture was inoculated in fresh medium and collected for infections at the next mid-log phase.

### 4.4. Infections and Treatment

For in vitro infections, bacteria were washed twice with PBS by centrifugation at 3500× *g* for 10 min at 4 °C and passed several times through a 27 G needle with syringe to obtain a single-cell suspension. Bacterial numbers were estimated by measurement of OD_580_, and bacteria were opsonized in 50% fresh autologous serum in RPMI for 45 min at RT. Human neutrophils were infected at an MOI of 3 for 2 h followed by washing by centrifugation and resuspension in RPMI supplemented with 10% FCS and 2 mM L-glutamine and incubated at 37 °C with 5% CO_2_.

Animals were aerosol-infected with the H37Rv strain at an infection dose of 100–150 CFU from frozen stocks with known CFU. Infection doses of animals were confirmed by CFU of organs at d1 after infection (see below). Infected animals were health-scored (see Supplementary Table S1) and weighed weekly or daily (25–35 dpi) and received oral treatment twice a day between day 25 and day 35 post infection (Supplementary Figure S2).

### 4.5. Necrosis Assays

Release of lactate dehydrogenase (LDH) was measured using a cytotoxicity detection kit (Roche, Mannheim, Germany) according to the manufacturer’s instructions. In short, 50 µL of cell culture supernatant or extracellular protein fraction was added to 50 µL reaction reagent and incubated for 30 min, and absorbance was determined at 490 nm. For human peripheral blood neutrophils in vitro, Sytox Green (Thermo Fisher Scientific, Waltham, MA, USA) was added to the culture medium (5 µM), and fluorescence was measured using a plate reader (Biotek, Santa Clara, CA, USA). The percental amount was calculated using 100% values from cells lysed with 0.5% Triton X-100.

### 4.6. Colony-Forming Unit Assay

After removal, organs were homogenized in WTA buffer, 4-log serial dilution in PBS containing 0.05% Tween 80 was prepared, and dilutions were plated on 7H11 Agar plates. Mycobacterial colonies were counted after 3–4 weeks of incubation at 37 °C.

### 4.7. Histology

Organs from infected mice were fixated overnight at 4 °C in 4% PFA and dehydrated in an increasing ethanol row before being embedded in paraffin blocks and sectioned (4 µm). Slides were de-paraffinized using xylol and decreasing concentrations of ethanol. For Ziehl–Neelsen stainings, carbolfuchsin was used, slides were heated three times, dipped in 5% HCL in 70% Ethanol, counter-stained for 1 min with 1:10 Methylene Blue in Aqua bidest., dehydrated using increasing concentrations of ethanol and Xylol, and embedded in Entellan.

For immunohistochemical detection of MPO, antigens were retrieved by steaming in citrate buffer for 30 min. Endogenous peroxidases were blocked by a 1% H_2_O_2_ solution for 10 min. Blocking and permeabilization were performed by incubation with 0.1% Triton-X, 3% BSA, 5% normal goat serum for 10 min. Anti-MPO antibody (1:150, Thermo Fisher Scientific, Waltham, MA, USA) was incubated overnight at 4 °C, secondary biotinylated antibody goat α-rabbit-F(ab)2 antibody (1:500, Jackson Immuno Research, Ely UK) for 45 min at RT followed by incubations with ABC peroxidase staining kit (Thermo Fisher Scientific, Waltham, MA, USA) and DAB-staining solution (Sigma-Aldrich, St. Louis, MO, USA). Hemalaun staining was performed as described before.

### 4.8. Flow Cytometry

Organs were incubated in RPMI medium containing liberase TL (50 mg/mL, Sigma-Aldrich, St. Louis, MO, USA) and DNase I (50 mg/mL, Sigma-AldrichSt. Louis, MO, USA) for 90 min at 37 °C, passed through a 100 µm cell strainer, and centrifuged for 5 min, 4 °C, 500× *g*. Erythrocytes were lysed by ammonium chloride buffer (5 min, RT). Cells were incubated for 10 min at RT in 50 µL Fc-blocking buffer, containing anti-CD16/32 (0.5 µg/100 µL) (Thermo Scientific, Waltham, MA, USA), 1% rat serum, and 1% Syrian hamster serum, followed by indicated antibody (CD3, CD4, CD8e, CD11c, CD49b, CD69 all 1:200; CD11b, Ly6G both 1:400; IFN-g, MHC-II, MPO, TNF-a all 1:400) (Biolegend, San Diego, CA, USA) incubation together with DHR123 (50 µM) (Thermo Scientific, Waltham, MA, USA) for ROS and/or 7-AAD (1 µg/mL) (Thermo Scientific, Waltham, MA, USA) for necrosis assessment for 15 min on ice. For intracellular cytokine stainings, cells were treated with 5 µg/mL Brefeldin A in RPMI buffer in an anti-CD3/CD28-coated (5 µg/mL each) (Biolegend, San Diego, CA, USA) plate for 4,5 h at 37 °C with 5% CO_2_ followed by antibody incubation. Cells were analyzed with a MACS Quant Analyzer 10 (BD, Heidelberg, Germany).

### 4.9. MPO Concentration and Activity Assays

Extracellular proteins were extracted as described before [52]. In short, organs were incubated with protein extraction buffer (0.35 M sucrose, 1 mM CaCl2, 10 U/mL Heparin in HBSS) for 2 h on ice. After centrifugation (5 min, 4 °C, 500× *g*), proteins in supernatants were precipitated with ice-cold acetone (1 h at −20 °C), followed by centrifugation at 3500× *g* for 15 min at 4 °C. Total protein concentrations were calculated in lung lysates, extracellular protein fractions, and sera according to the manufacturer’s protocol using the BCA-based Pierce 660 nm protein assay (Thermo Scientific, Waltham, MA, USA). To calculate MPO activity, reaction of MPO with 3,3′, 5,5′-Tetramethylbenzidin (TMB) was colorimetrically measured 10 min after incubation and stopping of the reaction by H_2_SO at 650 nm. To evaluate the concentration of MPO, the mouse myeloperoxidase DuoSet ELISA kit (R&D, UK) was used according to the manufacturer’s instructions. In short, ELISA plates were coated with 800 ng/mL capture anti-MPO antibody, blocked for 1 h with 1% BSA in PBS, incubated with samples for 2 h, followed by addition of detection antibody (50 ng/mL) for 2 h. Streptavidin–HRP solution was added for 20 min followed by 100 µL substrate solution for 15–20 min. Reaction was stopped using 2 M H_2_SO. Absorbance was acquired at 540 nm. Standards of recombinant MPO were used to calculate MPO concentrations.

### 4.10. Cytokine Measurement

For cytokine concentration analysis of lung lysates, multiplex immunoassays were used (LegendPlex mouse proinflammatory chemokine kit from Biolegend, San Diego, CA USA, or a customized U-Plex kit from MSD, Rockville, MD, USA) according to the manufacturer’s instructions. In short, in the bead-based LegendPlex assay, beats of different size and granularity were coated with specific capturing antibodies. Detection antibodies and secondary fluorescently labeled antibodies were used to detect cytokines and provided standards using a BD FACSCanto II flow cytometer. For the U-Plex MSD kit, biotinylated antibodies against each analyte were coupled with individual U-PLEX linkers that were coated on specific areas in a well plate. For detection, antibodies labeled with electrochemiluminescent labels (MSD GOLD SULFO-TAG) were used, and electricity was applied to stimulate light emission. Intensities were measured to calculate concentrations. For measurement, an MSD instrument was used.

### 4.11. Statistical Analysis

Statistical analysis was performed using GraphPad Prism. The analysis of more than two groups at one time point was performed using ordinary one-way ANOVA with Turkey’s multiple comparison post-test. An ordinary two-way ANOVA with Turkey’s multiple comparison post-test was used for experiments, comparing different groups with more time points. Statistical significance was reached with a *p* value of < 0.05. Significance is marked with stars * *p* ≤ 0.05; ** *p* ≤ 0.01; *** *p* ≤ 0.001.

## Data Availability

The data presented in this study are available on request from the corresponding author.

## Supplementary Materials

The following supporting information can be downloaded at: https://www.mdpi.com/article/10.3390/ijms23052554/s1.

## Abbreviations

ABAH	4-Aminobenzoic acid hydrazide
DHR123	dihydrorhodamine 123
INH	isoniazid
iNOS	inducible NO synthase
LDH	lactate dehydrogenase
MDR/XDR	multi and extensively drug-resistant
MPO	myeloperoxidase
ROS	reactive oxygen species
RNS	reactive nitrogen species

## References

[1] Adams L.B., Dinauer M.C., Morgenstern D.E., Krahenbuhl J.L. (1997). Comparison of the roles of reactive oxygen and nitrogen intermediates in the host response to *Mycobacterium tuberculosis* using transgenic mice. Tuber. Lung Dis..

[2] Baghaei P., Tabarsi P., Dorriz D., Marjani M., Shamaei M., Pooramiri M.V., Mansouri D., Farnia P., Masjedi M., Velayati A. (2011). Adverse effects of multidrug-resistant tuberculosis treatment with a standardized regimen: A report from Iran. Am. J. Ther..

[3] Balaiya S., Chalam K.V. (2014). An In vitro Assay to Quantify Nitrosative Component of Oxidative Stress. J. Mol. Genet. Med..

[4] Berry M.P., Graham C.M., Mcnab F.W., Xu Z., Bloch S.A., Oni T., Wilkinson K.A., Banchereau R., Skinner J., Wilkinson R.J. (2010). An interferon-inducible neutrophil-driven blood transcriptional signature in human tuberculosis. Nature.

[5] Bohrer A.C., Castro E., Hu Z., Queiroz A.T.L., Tocheny C.E., Assmann M., Sakai S., Nelson C., Baker P.J., Ma H. (2021). Eosinophils are part of the granulocyte response in tuberculosis and promote host resistance in mice. J. Exp. Med..

[6] Chen S., Chen H., Du Q., Shen J. (2020). Targeting Myeloperoxidase (MPO) Mediated Oxidative Stress and Inflammation for Reducing Brain Ischemia Injury: Potential Application of Natural Compounds. Front. Physiol..

[7] Corleis B., Korbel D., Wilson R., Bylund J., Chee R., Schaible U.E. (2012). Escape of *Mycobacterium tuberculosis* from oxidative killing by neutrophils. Cell Microbiol..

[8] Crow J.P. (1997). Dichlorodihydrofluorescein and dihydrorhodamine 123 are sensitive indicators of peroxynitrite in vitro: Implications for intracellular measurement of reactive nitrogen and oxygen species. Nitric Oxide.

[9] D’elia R.V., Harrison K., Oyston P.C., Lukaszewski R.A., Clark G.C. (2013). Targeting the “Cytokine Storm” for Therapeutic Benefit. Clin. Vaccine Immunol..

[10] Dallenga T., Linnemann L., Paudyal B., Repnik U., Griffiths G., Schaible U.E. (2018). Targeting neutrophils for host-directed therapy to treat tuberculosis. Int. J. Med. Microbiol..

[11] Dallenga T., Repnik U., Corleis B., Eich J., Reimer R., Griffiths G.W., Schaible U.E.M. (2017). tuberculosis-Induced Necrosis of Infected Neutrophils Promotes Bacterial Growth Following Phagocytosis by Macrophages. Cell Host Microbe.

[12] Dallenga T., Schaible U.E. (2016). Neutrophils in tuberculosis--first line of defence or booster of disease and targets for host-directed therapy?. Pathog. Dis..

[13] Demetri G.D., Griffin J.D. (1991). Granulocyte colony-stimulating factor and its receptor. Blood.

[14] Domaszewska T., Scheuermann L., Hahnke K., Mollenkopf H., Dorhoi A., Kaufmann S.H.E., Weiner J. (2017). Concordant and discordant gene expression patterns in mouse strains identify best-fit animal model for human tuberculosis. Sci. Rep..

[15] Driver E.R., Ryan G.J., Hoff D.R., Irwin S.M., Basaraba R.J., Kramnik I., Lenaerts A.J. (2012). Evaluation of a mouse model of necrotic granuloma formation using C3HeB/FeJ mice for testing of drugs against Mycobacterium tuberculosis. Antimicrob. Agents Chemother..

[16] Etna M.P., Giacomini E., Severa M., Coccia E.M. (2014). Pro- and anti-inflammatory cytokines in tuberculosis: A two-edged sword in TB pathogenesis. Semin. Immunol..

[17] Eum S.Y., Kong J.H., Hong M.S., Lee Y.J., Kim J.H., Hwang S.H., Cho S.N., Via L.E., Barry C.E. (2010). Neutrophils are the predominant infected phagocytic cells in the airways of patients with active pulmonary TB. Chest.

[18] Forghani R., Kim H.J., Wojtkiewicz G.R., Bure L., Wu Y., Hayase M., Wei Y., Zheng Y., Moskowitz M.A., Chen J.W. (2015). Myeloperoxidase propagates damage and is a potential therapeutic target for subacute stroke. J. Cereb. Blood Flow Metab..

[19] Forghani R., Wojtkiewicz G.R., Zhang Y., Seeburg D., Bautz B.R., Pulli B., Milewski A.R., Atkinson W.L., Iwamoto Y., Zhang E.R. (2012). Demyelinating diseases: Myeloperoxidase as an imaging biomarker and therapeutic target. Radiology.

[20] Hoffmann H., Kohl T.A., Hofmann-Thiel S., Merker M., Beckert P., Jaton K., Nedialkova L., Sahalchyk E., Rothe T., Keller P.M. (2016). Delamanid and Bedaquiline Resistance in *Mycobacterium tuberculosis* Ancestral Beijing Genotype Causing Extensively Drug-Resistant Tuberculosis in a Tibetan Refugee. Am. J. Respir. Crit. Care Med..

[21] Kim H., Wei Y., Lee J.Y., Wu Y., Zheng Y., Moskowitz M.A., Chen J.W. (2016). Myeloperoxidase Inhibition Increases Neurogenesis after Ischemic Stroke. J. Pharmacol. Exp. Ther..

[22] Kim H.J., Wei Y., Wojtkiewicz G.R., Lee J.Y., Moskowitz M.A., Chen J.W. (2019). Reducing myeloperoxidase activity decreases inflammation and increases cellular protection in ischemic stroke. J. Cereb. Blood Flow Metab..

[23] Kimmey J.M., Huynh J.P., Weiss L.A., Park S., Kambal A., Debnath J., Virgin H.W., Stallings C.L. (2015). Unique role for ATG5 in neutrophil-mediated immunopathology during *M. tuberculosis* infection. Nature.

[24] Kramnik I., Beamer G. (2016). Mouse models of human TB pathology: Roles in the analysis of necrosis and the development of host-directed therapies. Semin. Immunopathol..

[25] Macmicking J.D., North R.J., Lacourse R., Mudgett J.S., Shah S.K., Nathan C.F. (1997). Identification of nitric oxide synthase as a protective locus against tuberculosis. Proc. Natl. Acad. Sci. USA.

[26] Mckenzie S.J., Baker M.S., Buffinton G.D., Doe W.F. (1996). Evidence of oxidant-induced injury to epithelial cells during inflammatory bowel disease. J. Clin. Investig..

[27] Metcalf D. (1991). Control of granulocytes and macrophages: Molecular, cellular, and clinical aspects. Science.

[28] Misharin A.V., Morales-Nebreda L., Mutlu G.M., Budinger G.R., Perlman H. (2013). Flow cytometric analysis of macrophages and dendritic cell subsets in the mouse lung. Am. J. Respir. Cell Mol. Biol..

[29] Mishra B.B., Lovewell R.R., Olive A.J., Zhang G., Wang W., Eugenin E., Smith C.M., Phuah J.Y., Long J.E., Dubuke M.L. (2017). Nitric oxide prevents a pathogen-permissive granulocytic inflammation during tuberculosis. Nat. Microbiol..

[30] Moreira-Teixeira L., Stimpson P.J., Stavropoulos E., Hadebe S., Chakravarty P., Ioannou M., Aramburu I.V., Herbert E., Priestnall S.L., Suarez-Bonnet A. (2020). Type I IFN exacerbates disease in tuberculosis-susceptible mice by inducing neutrophil-mediated lung inflammation and NETosis. Nat. Commun..

[31] Moreira-Teixeira L., Tabone O., Graham C.M., Singhania A., Stavropoulos E., Redford P.S., Chakravarty P., Priestnall S.L., Suarez-Bonnet A., Herbert E. (2020). Mouse transcriptome reveals potential signatures of protection and pathogenesis in human tuberculosis. Nat. Immunol..

[32] Ohnishi S., Murata M., Kawanishi S. (2002). DNA damage induced by hypochlorite and hypobromite with reference to inflammation-associated carcinogenesis. Cancer Lett..

[33] Pacher P., Beckman J.S., Liaudet L. (2007). Nitric Oxide and Peroxynitrite in Health and Disease. Physiol. Rev..

[34] Parida S.K., Axelsson-Robertson R., Rao M.V., Singh N., Master I., Lutckii A., Keshavjee S., Andersson J., Zumla A., Maeurer M. (2015). Totally drug-resistant tuberculosis and adjunct therapies. J. Intern. Med..

[35] Parker H., Dragunow M., Hampton M.B., Kettle A.J., Winterbourn C.C. (2012). Requirements for NADPH oxidase and myeloperoxidase in neutrophil extracellular trap formation differ depending on the stimulus. J. Leukoc. Biol..

[36] Pulli B., Ali M., Forghani R., Schob S., Hsieh K.L., Wojtkiewicz G., Linnoila J.J., Chen J.W. (2013). Measuring myeloperoxidase activity in biological samples. PLoS ONE.

[37] Rausch P.G., Moore T.G. (1975). Granule enzymes of polymorphonuclear neutrophils: A phylogenetic comparison. Blood.

[38] Saini R., Singh S. (2019). Inducible nitric oxide synthase: An asset to neutrophils. J. Leukoc. Biol..

[39] Simms P.E., Ellis T.M. (1996). Utility of flow cytometric detection of CD69 expression as a rapid method for determining poly- and oligoclonal lymphocyte activation. Clin. Diagn. Lab. Immunol..

[40] Szabó C., Ischiropoulos H., Radi R. (2007). Peroxynitrite: Biochemistry, pathophysiology and development of therapeutics. Nat. Rev. Drug Discov..

[41] Ten R.M., Pease L.R., Mckean D.J., Bell M.P., Gleich G.J. (1989). Molecular cloning of the human eosinophil peroxidase. Evidence for the existence of a peroxidase multigene family. J. Exp. Med..

[42] Van Zandbergen G., Hermann N., Laufs H., Solbach W., Laskay T. (2002). Leishmania promastigotes release a granulocyte chemotactic factor and induce interleukin-8 release but inhibit gamma interferon-inducible protein 10 production by neutrophil granulocytes. Infect. Immun..

[43] Vilaplana C., Marzo E., Tapia G., Diaz J., Garcia V., Cardona P.J. (2013). Ibuprofen therapy resulted in significantly decreased tissue bacillary loads and increased survival in a new murine experimental model of active tuberculosis. J. Infect. Dis..

[44] Wang L., Taneja R., Razavi H.M., Law C., Gillis C., Mehta S. (2012). Specific role of neutrophil inducible nitric oxide synthase in murine sepsis-induced lung injury in vivo. Shock.

[45] Wheeler M.A., Smith S.D., García-Cardeña G., Nathan C.F., Weiss R.M., Sessa W.C. (1997). Bacterial infection induces nitric oxide synthase in human neutrophils. J. Clin. Investig..

[46] Whiteman M., Jenner A., Halliwell B. (1997). Hypochlorous acid-induced base modifications in isolated calf thymus DNA. Chem. Res. Toxicol..

[47] Whiteman M., Spencer J.P., Jenner A., Halliwell B. (1999). Hypochlorous acid-induced DNA base modification: Potentiation by nitrite: Biomarkers of DNA damage by reactive oxygen species. Biochem. Biophys. Res. Commun..

[48] WHO (2021). Global Tuberculosis Report 2021.

[49] Yang T.W., Park H.O., Jang H.N., Yang J.H., Kim S.H., Moon S.H., Byun J.H., Lee C.E., Kim J.W., Kang D.H. (2017). Side effects associated with the treatment of multidrug-resistant tuberculosis at a tuberculosis referral hospital in South Korea: A retrospective study. Medicine.

[50] Yeremeev V., Linge I., Kondratieva T., Apt A. (2015). Neutrophils exacerbate tuberculosis infection in genetically susceptible mice. Tuberculosis.

[51] Yousefi S., Gold J.A., Andina N., Lee J.J., Kelly A.M., Kozlowski E., Schmid I., Straumann A., Reichenbach J., Gleich G.J. (2008). Catapult-like release of mitochondrial DNA by eosinophils contributes to antibacterial defense. Nat. Med..

[52] Zhang H., Ray A., Miller N.M., Hartwig D., Pritchard K.A., Dittel B.N. (2016). Inhibition of myeloperoxidase at the peak of experimental autoimmune encephalomyelitis restores blood-brain barrier integrity and ameliorates disease severity. J. Neurochem..

