# Supplementary material for: Evaluation of Myeloperoxidase as Target for Host-Directed Therapy in Tuberculosis In Vivo
Source: Int J Mol Sci. 2022 Feb 25;23(5):2554. doi: 10.3390/ijms23052554 (PMC8910451; doi:10.3390/ijms23052554)
Supplement: Supplementary file 1 [file ijms-23-02554-s001.zip › suppl table 1.pdf]

Score	Activity	Bodyweight	General conditions	Behaviour
1	Very active	No change or increase	Fur glossy and glowing, clean orifices of the body, eyes clear and glossy	Normal
2	Active	Loss of < 10%	Reduced or abnormal body hygiene, uneven fur	Little changes
3	Less active	Loss of 10 - 20%	Dull, unkempt fur, unkempt orifices, elevated tonicity	Unusual, reduced motor functions or hyperkinetic
4	Not active	Loss of 20 - 30%	Dirty fur, clotted orifices, abnormal body position, eyes cloudy, elevated tonicity	Self-isolation, lethargic, pronounced hyperkinetic, coordination disorder, behavioral stereotypes
5	lethargic	Loss of > 30%	Cramps, paralysis in extremities and torso, breathing noises, cold body	Pain during handling, self-amputation (auto aggression)

Suppl. Table S1: Scoring system. After aerosol infection with ~100-150 CFU of H37Rv, mice were scored daily from day 25-35 after infection.
